# Spontaneous Formation of Eutectic Crystal Structures in Binary and Ternary Charged Colloids due to Depletion Attraction

**DOI:** 10.1038/srep23292

**Published:** 2016-03-17

**Authors:** Akiko Toyotama, Tohru Okuzono, Junpei Yamanaka

**Affiliations:** 1 Faculty of Pharmaceutical Sciences, Graduate School of Nagoya City University, 3-1 Tanabe, Mizuho, Nagoya, Aichi 467-8603, Japan; 2 JST, PRESTO, 4-1-8 Honcho, Kawaguch, Saitama, 332-0012, Japan

## Abstract

Crystallization of colloids has extensively been studied for past few decades as models to study phase transition in general. Recently, complex crystal structures in multi-component colloids, including alloy and eutectic structures, have attracted considerable attention. However, the fabrication of 2D area-filling colloidal eutectics has not been reported till date. Here, we report formation of eutectic structures in binary and ternary aqueous colloids due to depletion attraction. We used charged particles + linear polyelectrolyte systems, in which the interparticle interaction could be represented as a sum of the electrostatic, depletion, and van der Waals forces. The interaction was tunable at a lengthscale accessible to direct observation by optical microscopy. The eutectic structures were formed because of interplay of crystallization of constituent components and accompanying fractionation. An observed binary phase diagram, defined by a mixing ratio and inverse area fraction of the particles, was analogous to that for atomic and molecular eutectic systems. This new method also allows the adjustment of both the number and wavelengths of Bragg diffraction peaks. Furthermore, these eutectic structures could be immobilized in polymer gel to produce self-standing materials. The present findings will be useful in the design of the optical properties of colloidal crystals.

Self-organizations of colloidal particles in dispersions, i.e., crystallization^1^,^2^,^3^,^4^,^5^,^6^,^7^,^8^,^9^,^10^ and clustering^11^,^12^,^13^,^14^,^15^,^16^,^17^,^18^, produce ordered arrangements of the particles. In multicomponent colloids, these orderings create more complex structures; they include alloys (superlattices^19^,^20^,^21^,^22^ and eutectics^23^,^24^) in size-asymmetric binary colloids, and also heterogeneous clusters of well-defined shapes^11^,^16^,^18^. These complex structures will be useful as models of their atomic and molecular analogues, as well as novel materials in, e.g., photonics^25^,^26^. Here, we report formation of two dimensional (2D) area-filling colloidal eutectic structures in binary and ternary colloids. These eutectic structures — homogeneous mixtures of crystals of each component — were spontaneously formed because of interplay of crystallization of constituent components and accompanying fractionations.

Colloidal crystals are ordered structures of submicron-sized uniform particles in two- or three dimensions^1^,^2^,^3^,^4^,^5^,^6^,^7^,^8^,^9^,^10^. Over the past few decades, the colloidal crystallization has extensively been studied as models to study phase transition in general^1^,^2^,^3^,^5^,^6^. Their structures significantly vary depending on kind of interaction between the particles. Hard sphere (HS) colloids^1^,^2^, where the particles interact only via HS repulsion, crystallize at the particle volume fraction (*ϕ*) of approximately 0.49 (Alder transition). Closely packed opal structures are formed at *ϕ* = 0.74 in the HS colloids. For charged particles, electrostatic interactions lead to the formation of ordered crystals even at much lower *ϕ* 2,5,6,7,8,10.

Other important interactions for structural formations include depletion attraction that arises from the presence of non-adsorbing depletants in the medium^27^,^28^,^29^. Frequently, linear polymers are used as the depletant. An illustration of the depletion interaction is shown in Fig. 1(a). The polymer chain present in the medium cannot enter the gap between the particles, which is narrower than the polymer chain size in a solution (red-colored region); such a gap is called the depletion zone. The resulting difference between the polymer concentrations in the bulk and depletion regions causes a difference in the osmotic pressures, Δ*p*, leading to an attraction between the particles. Colloidal crystals were formed under sufficiently high particle and/or polymer concentrations^29^. When Δ*p* is sufficiently high, even charged colloids interacting via electrostatic repulsion crystalize due to the depletion force. Kose and Hachisu^4^ have reported that the depletion attraction-induced crystallization of charged polystyrene (PS) particles coexisting with the charged sodium polyacrylate (NaPAA) polymer. In the present study, we also use this system for the crystallization. We demonstrate that the interparticle interaction in the present system can be represented as a sum of the electrostatic, depletion, and van der Waals forces. By controlling over these competing interactions, we could tune the interaction at a lengthscale accessible to direct observation by optical microscopy.

Thus far, several authors have reported formations of eutectics in non-charged colloids. In binary hard sphere colloids subjecting gravitational sedimentation, fluid-crystal phase separation has been observed, and the presence of eutectics was also predicted theoretically^30^. Kozina *et al.*^23^,^24^ have recently reported the formation of eutectic structures in density-matched binary colloids. They determined precise phase diagram of the eutectics formation, by applying light scattering. However, 2D area-filling colloidal eutectics have not been reported till date.

Colloidal crystals have also been investigated as potential photonic materials^25^, because their Bragg wavelengths *λ*_B_ can be set in visible to near infrared regions. *λ*_B_ is given by the Bragg relation 2*d* sin *θ* = *λ*_B_/*n*_r_, where *d* is the distance between the crystal planes, *θ* is the incident angle of the light, and *n*_r_ is the refraction index of the sample. The eutectic colloidal crystals should have multiple Bragg peaks, whose *λ*_B_ values and intensities are easily tunable by changing the sizes and concentrations of the each constituent. These eutectic structures should have showed more advanced optical properties if area–filling materials are obtained. Here, we report the fabrications of binary and ternary eutectic colloidal crystals that filled the bottom plane of the containers. These structures were formed owing to the depletion force and sedimentation of the particles. The crystal lattice planes were well oriented parallel to the container bottom planes, facilitating the control of the optical properties. We also report immobilization of the eutectic structures in polymer gel matrix.

## Results and Discussion

### Depletion-induced crystallization in one-component PS + NaPAA

The characteristics of the particles we used are listed in Table 1. The diameters and polydisersity indexes (p.d.) of the particles were determined by dynamic light scattering (DLS) measurements. The zeta potentials *ζ* of the particles were estimated from electrostatic mobility determined by microscopic electrophoresis. The NaAA was prepared by adding NaOH to polyacrylic acid solution, until the degree of neutralization was 0.5. The weight average molecular weight, *M*_w_, of NaPAA was 8.2 × 10^5^ (degree of polymerization = 8.7 × 10^3^), and polydispersity index, *M*_w_/*M*_n_ (*M*_n_ is the number average molecular weight) was 3.2. The radius of gyration *R*_g_ of NaPAA in water, obtained by DLS, was 250 nm. Ratios of the particle radius *a*_p_ to *R*_g_ (≡ *q*) were also shown in Table 1. The overlap concentration of polymer *n*_p_* = 3/(4π*R*_g_^3^) was estimated to be 1.53  × 10^13^/cm^3^ (*C*_p_* = 10^3^*M*_w_ (*n*_p_*/*N*_A_) = 1.8 × 10^−3^ wt%; *N*_A_ is the Avogadro constant). All the experiments was performed at *C*_p_ = 0.08 wt%, that is, *C*_p_/*C*_p_* ~ 44. The effective charge number *Z*_eff_ of a NaPAA molecule, determined by means of conductivity measurements, was 2200. More details on the characterization will be described in Method section.

**Figure 1:**
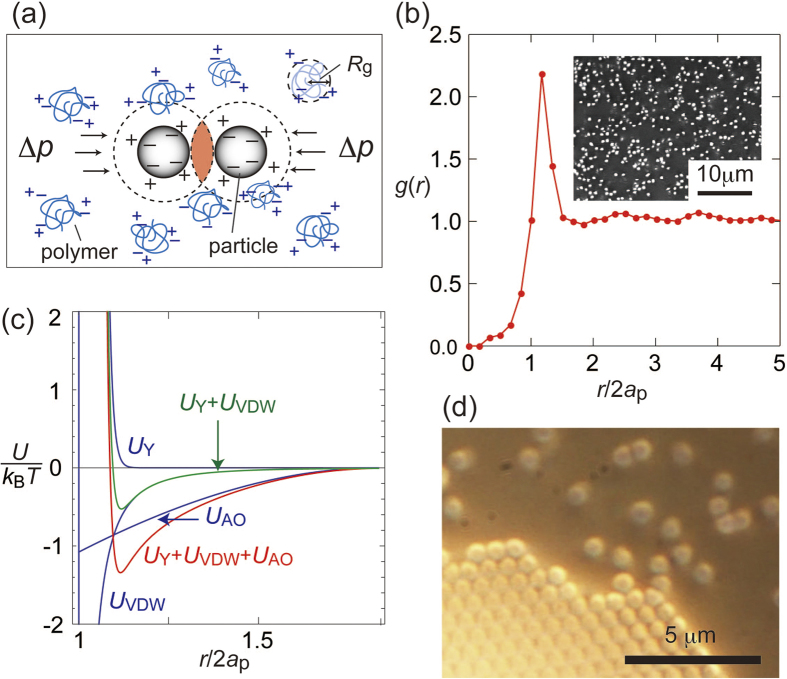
(**a**) An illustration of depletion attraction between the two charged colloidal particles owing to linear polymers (polyelectrolytes, in the present study). (**b**) A micrograph for the fluid state of PS600 + sodium polyacrylates(NaPAA) dispersion (inset; *C*_p_ = 0.08 wt%, *ϕ* = 6.7 × 10^−3^), and the radial distribution function *g*(*r*). (**c**) Interaction potential between the particles. (**d**) Optical micrograph of crystal–fluid interface (PS600, *C*_p_ = 0.08 wt%.).

We first examined the depletion interaction in one-component PS + NaPAA systems in disordered fluid state. Figure 1(b) shows a 2D radial distribution function of the particle, *g*(*r*) (*r* is the center-to-center distance of the particles), for PS600 (*ϕ* = 6.7 × 10^−3^) + NaPAA colloid, determined from a micrograph (shown in inset). The particle number concentration *n* = 3/(4π*a*_p_^3^) = 8.8 × 10^12^ cm^−3^. The *g*(*r*) had a first maximum at *r*/2*a*_p_ = 1.16. That is, at the closest approach, the particles were not in contact to each other but located with a separation of 0.32*a*_p_ (~100 nm) on average. This suggests that the depletion attraction overcame the electrostatic repulsion between the particles at the short distance.

We calculated a pair interaction potential of the particles, in terms of the electrostatic, van der Waals (VDW), and depletion potentials as follows. When the surface potential of the particle is sufficiently low, the electrostatic interaction is often represented by Yukawa potential^2^, *U*_Y_(*r*):


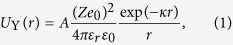


where *Z* is the particle charge number and *e*_0_ is the elementary charge; *ε*_r_ and *ε*_0_ the relative permittivity of medium and vacuum permittivity. *κ* is Debye parameter defined as *κ*^2^ = *ε*_0_^2^*I*/(*ε*_r_*ε*_0_*k*_B_*T*), where *I* is the ionic strength. The correction factor for the particle size is given by *A* = exp(2*κa*_p_)/(1 + *κa*_p_)^2^. By performing the electrical conductivity measurements, we determined *Z* of PS600 was 1.33 × 10^4^. The values of *I* and *κ* calculated from *Z*_eff_ of NaPAA and *C*_p_ were 2.17 mM and 6.5 nm, respectively.

The VDW potential between two spheres^2^ is given by


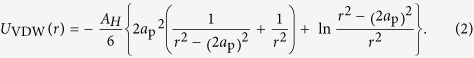


Here *A*_H_ is the Hamaker constant; for PS/water/PS system, *A*_H_ is estimated to be 4.75 *k*_B_*T* (*k*_B_ is the Boltzmann constant and *T* the temperature) by applying Lifsitz theory^31^.

The interaction potential for the depletion attraction, *U*_AO_(*r*), has derived by Asakura-Oosawa^27^ and Vrij^32^:


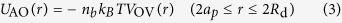



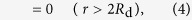


where *R*_d_ = *a*_p_ + *R*_g_ is the depletion radius, and *V*_OV_(*r*) is the overlap volume of spheres having radius of *R*_d_.


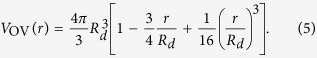


For example, for *a*_p_ = 300 nm and *C*_p_ = 0.08 wt%, we have *U*_AO_ = −1.08 *k*_B_*T* at *r* = 2*a*_p_.

In Fig. 1(c), *U*_Y_, *U*_VDW_, and *U*_AO_ were represented by blue solid curves, while *U*_Y_ + *U*_VDW_ and total potential interaction *U* = *U*_Y_ + *U*_VDW_ + *U*_AO_ was shown in green and red, respectively. It is clear that *U* (*r*) had a minimum (~ −1 *k*_B_*T*) at *r*/2*a*_p_ = 1.12, which was in a close agreement with the closet distance for *g*(*r*). Thus, under the present conditions, we can assume that two PS particles interact via net attraction. We expect that this system provides a useful model to study the phase behavior in a single-particle-level. We would like to note, however, that both NaPAA and PS particles have large numbers of counterions, which enhances the osmotic pressure in the medium. A difference in the small ion concentrations in bulk and depletion zone, if any, should cause additional attraction/repulsion. Further experimental study on this respect is in progress.

The PS particles settled to the bottom of the cell in a few days and formed crystal structures. High ionic strength in the colloids due to the NaPAA may have assisted the sedimentation. The crystal lattice planes were oriented parallel to the bottom of the container. Figure 1(d) shows a micrograph of the crystal/fluid (non-crystal) interface. A movie of the crystal growth process is shown as Supplementary Information S1 and S2. Here we can observe the motion of the particles near the interface. Microscopic observation of the crystal structure in the *z*-direction confirmed that the crystal planes comprised more than five layers.

### Observation of the eutectic formation process

**Figure 2:**
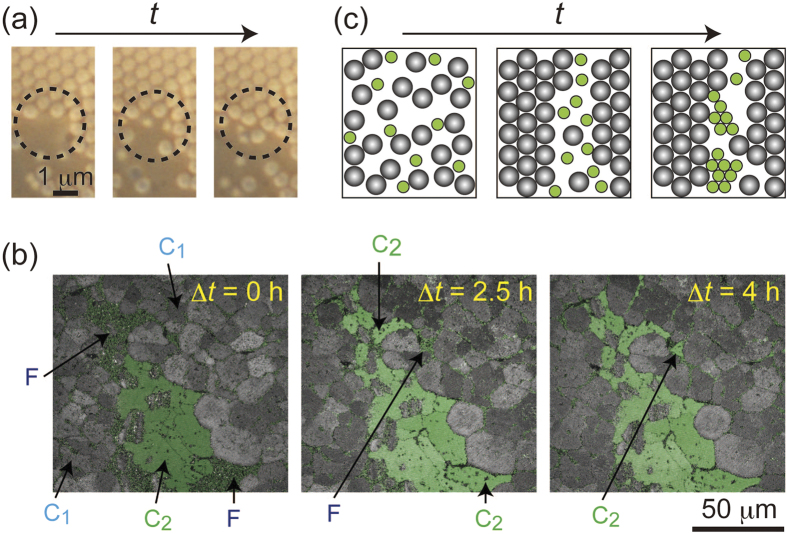
(**a**) Exclusion process of impurity particles at crystal–fluid interface (G300/PS600, particle size ratio = 1/1.8). (**b**) Crystal growth process of G500 in eutectic structure of binary (G500/PS600, particle size ratio = 1/1.2) colloid. (**c**) An illustration of the eutectic formation process.

We then examined the phase behavior of the binary colloids. We have already reported that the impurity particles were excluded from the crystals to the grain boundaries during grain growth in charged colloids^10^. Nozawa *et al.* have studied the impurity distribution in charged colloids based on the crystallization theory^33^. A similar exclusion behavior was observed for the present crystallization (Fig. 2(a), sample: G300/PS600 + NaPAA. *ϕ*  = 1.3 × 10^−4^ and 6.7 × 10^−3^, respectively). At first, vacancy sites were present at the crystal–fluid interface (left). Then, two small particles entered these sites but they were not trapped there (middle). On the other hand, particles with the equal size were incorporated into the crystal (right). That is a spontaneous fractionation of the particles occurred. A movie of these processes is shown in Supplementary Information S3. Size fractionation due to the depletion-induced crystallization has thus far been reported for particles with a broad size distribution^34^.

The abovementioned size fractionation in binary colloids resulted in eutectic structures, under suitable conditions. Figure 2(b) presents confocal laser scanning microscope (LSM) images showing the time evolution of the crystal structures in G500/PS600 binary colloid (*ϕ*  = 2.00 × 10^−2^ and 1.99 × 10^−2^, respectively) on the day 13 after preparation. Δ*t* represents the time course. Here we showed superposed micrographs taken by reflection and fluorescent modes. G500 and PS600 particles are shown as green-colored and monochrome images. At Δ*t* = 0, both the PS600 and G500 particles formed polycrystal structures (indicated as C1 and C2) and both of them were also partly presented in fluid region. At Δ*t* = 2.5 h, G500 particles were excluded during crystallization of PS600 and the accumulated G500 particles crystallized in between the crystal regions of PS600. At Δ*t* = 2.5 h, most of the particles, except those in the grain boundaries, form eutectic crystals. This mechanism of eutectic formation is illustrated in Fig. 2(c).

### Phase diagram of binary PS colloid

We determined the phase diagram of binary PS particles (PS600, G500) + NaPAA systems by using LSM. Though we prepared dilute samples (*ϕ* ~ 0.02), the particles settled out to the bottom of the container and crystallized. We observed the samples in both reflection and fluorescence modes to distinguish the two components. The area fractions of each component (*ϕ*_A1_ and *ϕ*_A2_) were determined from the micrographs.

**Figure 3:**
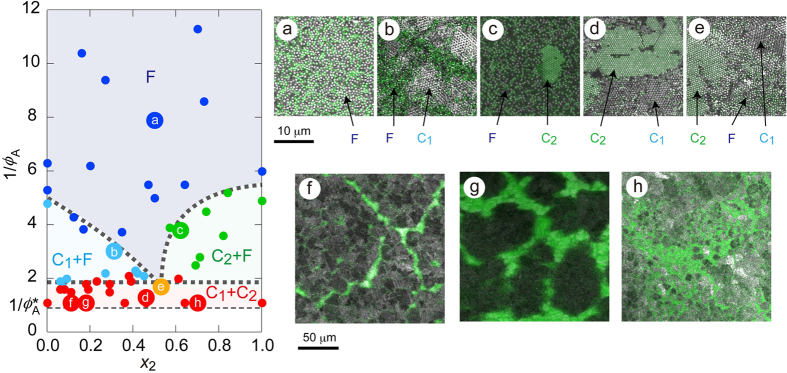
Phase diagram of binary colloids (G500/PS600, particle size ratio = 1/1.2, *C*_p_ = 0.08 wt%.) colloids defined by inverse of area fraction, 1/*ϕ*_A_, and volume fraction of G500, *X*_2_. The regions indicated F, C_1_, and C_2_ represent fluid phase, crystal phase of PS600 particles, and crystal phase of G500 particles, respectively. The LSM images of the samples shown (a ~ h) in the diagram are also shown.

The observed phase diagram is shown in Fig. 3. Usually, a binary phase diagram for atomic system plots temperature *T* against relative concentration of one of the constituents. For simplicity, here, we used 1/*ϕ*_A_ (*ϕ*_A_ = *ϕ*_A1_ + *ϕ*_A2_) as an ordinate of the phase diagram. The reduced pressure is also a useful parameter to describe the phase behavior of colloids^35^. The fraction of G500, *X*_2_ (=*ϕ*_A2_/*ϕ*_A_) is used as the abscissa in Fig. 3. Typical micrographs for various states in the phase diagram (a–h) are also shown in Fig. 3. It is clearly seen that the states of the colloids were classified into fluid, fluid + crystal of one of the components, and eutectic crystals. The eutectic region was present in 1/*ϕ*_A_ ≤ approximately 2 (*ϕ*_A_ = *ϕ*_A, e_ = 0.5), at all the *X*_2_ examined. Fluid/eutectic coexistence was observed at (1/*ϕ*_A_, *X*_2_) = (1.7, 0.53). The *ϕ*_A_ value at the closest packing, *ϕ*_A_*, is 0.91 (1/*ϕ*_A_* = 1.1). This implies that in *ϕ*_A, e_ ≤ *ϕ*_A_ ≤ *ϕ*_A_*, the particles were not in contact to each other, because of electrostatic repulsion. The present phase diagram is in analogous to binary phase diagrams of atomic eutectic systems^36^.

It appears to be of interest to know if the present systems can form “solid solution”; that is, crystal (solid) phase can dissolve single particles of another constituent. We sometimes observed that the crystals of one constituent included small numbers of another component. However, at the present stage of study, we could not distinguish if these states were equilibrium solid solution, or non-equilibrium structures. Further studies are in progress. We would also like to note that an incubation time for nucleation of one constituent in the eutectic phase, significantly varied depending on *X*_2_, and took the largest value at *X*_2_ ~ 0.5.

### Eutectic crystal in ternary PS colloid

**Figure 4:**
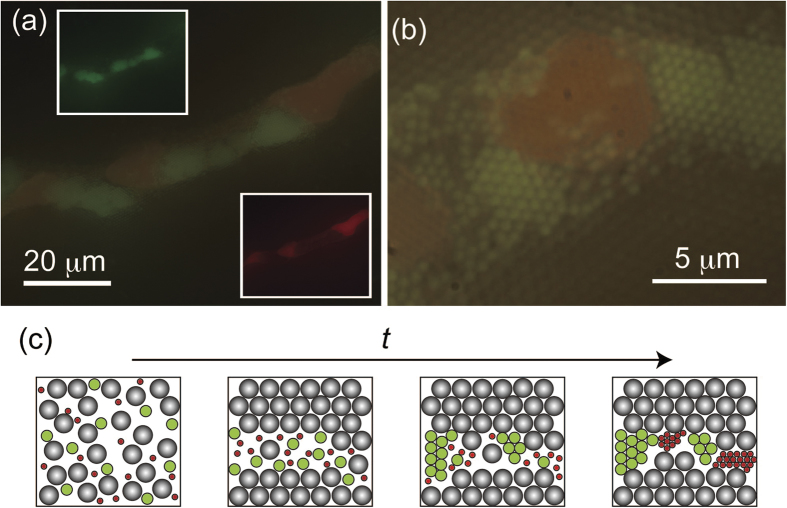
Optical micrographs of the eutectic crystal structure of ternary (DR390/G500/PS600) colloid. particle size ratio = 1/1.5/1.8. *C*_p_ = 0.08 wt%. (**a**) superimposed images of two crystals (inset) (**b**) enlarged image (**c**) Illustration for the formation of ternary eutectic structure.

Figure 4(a) shows the eutectic crystal structure of the ternary colloids comprising DR390, G500, PS600 (*ϕ*_i_ = 1.7 × 10^−3^, 8.0 × 10^−4^, 6.7 × 10^−3^). The insets in Fig. 4 show the superimposed images of the DR390 (right) and G500 (left) obtained using different optical filters. The PS600 particles are present in the dark regions of the image. Figure 4(b) is a magnified image showing opal structure of PS600 particles illuminated by the surrounding bright red and green particles. Using the Fourier transform of the images, we confirmed that the crystal structures had six-fold symmetries, implying that each component is spontaneously fractionated and arranged into an ordered crystal structure. As described above, we observed the successive fractionation and crystallization processes (Fig. 4(c)). Upon crystallization of the largest particles, the other components are excluded from the crystal boundary. This is followed by the successive crystallizations of the second and third components. A similar successive crystallization has been reported for eutectic formation in atomic and molecular systems^37^. We note that the crystallization is facilitated in constraint geometry^38^, which has a close relevance to the present finding.

### Optical property of the eutectic colloidal crystals

**Figure 5:**
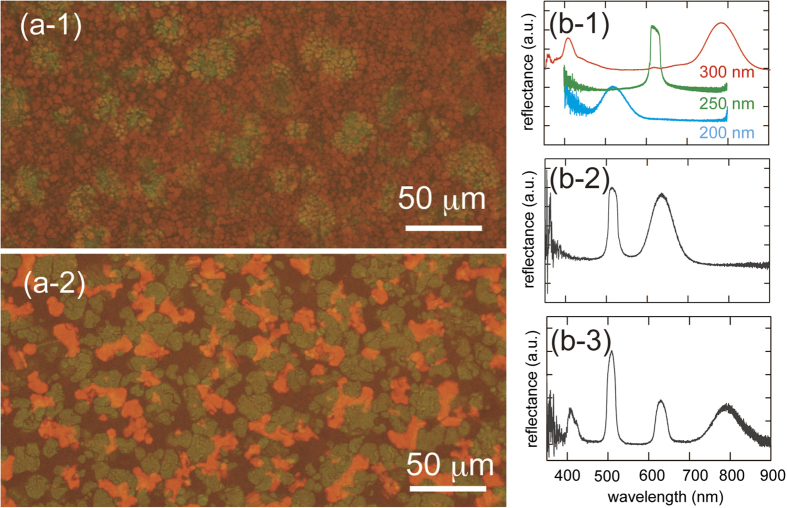
(**a**) Optical micrographs of (1) binary (PS200/PS250, particle size ratio = 1/1.1), and (2) ternary(PS200/PS250/PS300), particle size ratio = 1/1.1/1.4.) eutectic crystals; (**b**) reflectance spectra of (1) single component, (2) binary component, and (**c**) ternary eutectic crystals.

Using smaller particles, we then examined eutectic structures with Bragg wavelengths in the visible light region of the spectrum. Figure 5(a-1,2) show micrographs of the eutectic structures of binary and ternary colloids. The crystal grains exhibited two or three types of colored regions. Since the particles used here are not dyed with fluorescent molecules, the observed colors should be because of the Bragg diffraction from the crystal lattices. The grain size differences in these binary and ternary eutectics may be caused by the differences in the densities of the crystallization nuclei. Figure 5(b-1) shows the reflectance spectra of single-component crystals of PS200, PS250, and PS300. The spectra of the PS200 and PS250 crystals exhibited a single Bragg peak, while the PS300 crystal exhibited two peaks at approximately 780 and 400 nm, respectively. We calculated the Bragg wavelengths by assuming that the crystal lattice had face centered cubic lattice symmetry and the (111) planes of the obtained crystal are oriented parallel to the bottom of the cell. For this calculation, we used the volume average of refraction index *n*_r_ values for the PS particle (i.e., 1.59), and water (i.e., 1.33) and *ϕ* = 0.74 for PS. The calculated diffraction wavelengths are 533, 620, and 786 nm for PS200, PS250, and PS300, respectively, which are in good agreement with the observed peak wavelength (We note that these corresponds to the wavelengths of blue, green red lights. Thus, the present eutectic crystals will be useful as RGB optical filters). The multi-component systems are comprised PS 200 and PS 250 (*ϕ*_i_ = 0.010, 0.033) and PS 200, PS250, and PS300 (*ϕ*_i_ = 0.020, 0.0152, 0.0172). The reflection spectra of the binary and ternary colloids are shown in Fig. 5(b-2,3). The observed multiple peaks are due to the overlap of the spectra of the crystals of each single component. The formation process of the eutectic crystals was similar to that shown in Figs 2 and 4. In ternary colloids, the appearance of the peak due to the PS200 crystal was followed by the successive appearance of the PS250 and PS300 peaks.

### Immobilization of eutectic in polymer gel matrix

**Figure 6:**
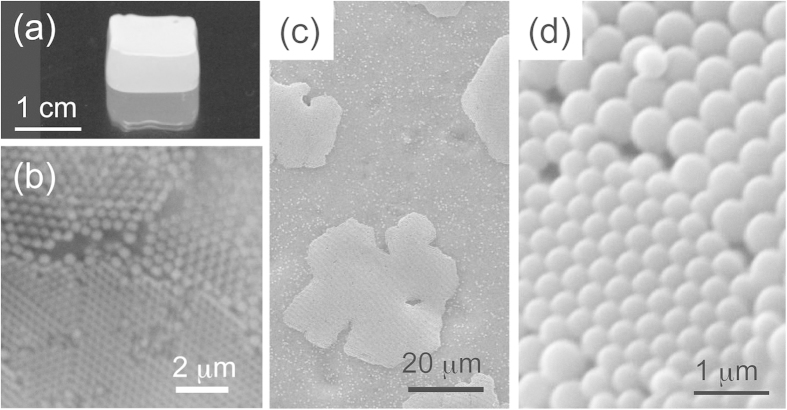
(**a**) Overview of the gelled sample (the sample was set upside down so that the sedimented layer was on the top surface). (**b**) Optical micrograph of binary (PS430 /PS600, particle size ratio = 1:1.4.) eutectic. (**c,d**) SEM images obtained during the crystal growth process and eutectic structure of binary (PS430/ PS600) colloid.

The obtained eutectic crystal structures could be immobilized in poly (*N*-methyrolacrylamide) polymer hydrogel using a slight modification of our previously reported photopolymerisation method^39^. We thus obtained self-standing eutectic materials. Figure 6(a) shows an image of the inverted gelled sample. The sample has a white turbid colored dense bottom. Here, the gelled sample was placed upside down, so that the top region in the image was formed by the sedimentation of the colloidal particles. An optical micrograph of the gelled eutectic structures and the detailed structure of these gelled samples during the crystal growth process (Fig. 6(c)) and obtained eutectic structure (Fig. 6(d)) are shown in Fig. 6(b,d). We expect that these immobilized crystals may find future applications as optical filters whose transmission and reflection characteristics are tunable by mechanical stress.

## Conclusion

We have demonstrated a spontaneous formation of eutectic crystals in binary and ternary colloids that oriented to the bottom of the container. We used charged PS particles + sodium polyacrylates, by which we could control interparticle interaction over competing electrostatic, depletion and van der Waals interactions. The eutectic crystal formation was attributed to a combination of depletion force and spontaneous fractionation during crystallization. We determined a binary phase diagram for two PS particles having different sizes, which were analogous to that for atomic eutectic system. We expect that our findings will be useful in the design of the optical properties of colloidal materials. Such colloids are also valuable as models for the general study of the eutectic structure formation process.

## Method

### Materials

We used the aqueous dispersions of the polystyrene particles of various diameters. The particles except PS250 were purchased from Thermo Scientific, MA., while PS250 was synthesized by emulsifier-free radical polymerization in our laboratory^40^.

Polyacrylic acid sample was purchased from Wako Chemicals, Tokyo, Japan. We determined the molecular weight and polydispersity of PAA as follows. It is known that the average molecular weight determined by viscosity measurements at theta condition is equal to *M*_w_. In the case of NaPAA, the theta condition of polymer in aqueous solution lay at [NaBr] = 1.5 M and *T* = 15 °C. The measured limiting viscosity [η] was 1.49 dl/g, which gave *M*_w_ = 8.18 × 10^5^ (*M*_w_ = 7.8 × 10^5^, for degree of neutralization, DN = 0.5). *M*_n_, of NaPAA (DN = 1) was determined by the osmotic pressure measurements using membrane osmometer under dilute condition (0.05 wt%). By using van’t Hoff relation, we determined *M*_n_ = 2.57 × 10^5^. The conductivity and pH titrations of PAA are described in Supplementary Information S4.

The hydrodynamic radius *R*_h_ of the NaPAA (DN = 0.5) in pure water, determined by performing dynamic light scattering (DLS) measurements, was 250 nm. Here we assumed this value as *R*_g_. Calculated *R*_g_ value for the PAA under theta condition [Gaussian coil, *R*_g_ = *b* (DP/6)^1/2^; *b* is the segment length = 0.252 nm, and degree of polymerization DP = 8.7 × 10^3^] is 12 nm. On the other hand, fully stretched length of the PAA is estimated as 2200 nm. Therefore under the present conditions, the PAA chain had rather extended conformation.

### Optical microscope observation

We observed the crystallization processes using glass-bottomed cells via an inverted microscope (Nikon, ECLIPSE-Ti, Japan) and confocal laser scanning microscope (Nikon, C2, Japan).

### Spectrometry

A multichannel spectrometer (type USB-2000, Ocean Optics Inc., Dunedin, FL) equipped with optical fiber probes was used for the right angle reflection spectra measurements.

### Immobilization by polymer gel

The reaction solutions were composed of 0.67 M *N*-methyrol acrylamide (gel monomer), 10 mM *N*,*N*’-methylenebisacrylamide (crosslinker), and 0.05 mg/ml 2,2′-azobis[2-methyl-*N*-[2-hydroxyethyl]-propionamide] (photo-induced radical polymerization initiator). After the solutions were deionized using ion-exchange resin beads, argon gas was bubbled through them to remove dissolved oxygen and carbon dioxide, which would otherwise inhibit the radical polymerization reaction. Before formation of the eutectic crystals, the abovementioned gellation reagents were dissolved in the multi-components colloid and polymer. Since the eutectic colloidal crystals were grown at the bottom of the cell, UV was illuminated from the bottom.

### SEM imaging

Gelled colloidal crystal could be observed by a scanning electron microscope (JEOL, JCM-6000, Tokyo, Japan) after treatment by ethanol for approximately 10 min. Using this process, a fraction of the polymer networks shrunk and the particles were exposed to external environment. We noted that since the obtained crystals had an opal structure, the shrinking of the gel during the processing by ethanol did not reduce the distances between the particles in the crystals.

## Additional Information

**How to cite this article**: Toyotama, A. *et al.* Spontaneous Formation of Eutectic Crystal Structures in Binary and Ternary Charged Colloids due to Depletion Attraction. *Sci. Rep.* **6**, 23292; doi: 10.1038/srep23292 (2016).

## Supplementary Material

Supplementary Information S1

Supplementary Information S2

Supplementary Information S3

Supplementary Information S4

## Figures and Tables

**Table 1:** Characteristics of the particles.

Particle	PS600	G500	PS430	DR390	G300	PS300	PS250	PS200
diameter (nm)	597	499	412	457	333	312	245	216
p.d. (%)	6.0	3.7	4.0	10.7	4.9	3.0	15.8	4.7
*ζ (mV)*	−60.7	−55.1	−34.7	−63.2	−75.2	−60.7	−79.6	−42.6
*q* (=*a*_p_/*R*_g_)	1.19	1.00	0.82	0.91	0.67	0.62	0.49	0.43

(G and DR in the particle names indicate green- and red-fluorescent dyed particles, respectively. The values of *ζ* were obtained by Henry’s equation from the observed electrophoretic mobility).

## References

[b1] Bartlett P. & van Megen W. In Metha A. (ed), Granular Matter (Springer, New York, 195–257, 1994).

[b2] Russel W. B., Saville D. A. & Schowalter W. R. Colloidal Dispersions (Cambridge University Press: New York, 1989).

[b3] Sood A. K. Solid State Phys., [Eds Ehrenreich H. & Turnbull D. ] (Academic Press, New York, 1991).

[b4] Kose A. & Hachisu S. Ordered structure in weakly flocculated monodisperse latex. J. Colloid Interface Sci. 55, 487–498 (1976).

[b5] Pieranski P. Colloidal crystals. Contemp. Phys. 24, 25–73 (1983).

[b6] Anderson V. J. & Lekkerkerker H. N. W. Insights into phase transition kinetics from colloid science. Nature 416, 811–815 (2002). 11976674 10.1038/416811a

[b7] van Blaaderen A. Colloids under External Control. MRS Bull. 29, 85–90 (2004).

[b8] Yethiraj A. & van Blaaderen A. A colloidal model system with an interaction tunable from hard sphere to soft dipolar. Nature 421, 513–517 (2003). 12556887 10.1038/nature01328

[b9] Stipp A. *et al.* Optical experiments on a crystallizing hard-sphere–polymer mixture at coexistence. Phys. Rev. E 81, 051401 (2010). 10.1103/PhysRevE.81.051401 20866224

[b10] Yoshizawa K., Toyotama A., Okuzono T. & Yamanaka J. Exclusion of impurity particles in charged colloidal crystals. Soft Matter 10, 3357–3361 (2014). 24807633 10.1039/c3sm52912f

[b11] Manoharan V. N., Elsesser M. T. & Pine D. J. Dense Packing and Symmetry in Small Clusters of Microspheres. Science 301, 483–487 (2003). 12881563 10.1126/science.1086189

[b12] van Blaaderen A. Colloids get complex. Nature 439, 545–546 (2006). 16452966 10.1038/439545a

[b13] Meng G., Arkus N., Brenner M. P. & Manoharan V. N. The Free-Energy Landscape of Clusters of Attractive Hard Spheres. Science 327, 560–563 (2010). 20110500 10.1126/science.1181263

[b14] Wang Y. *et al.* Colloids with valence and specific directional bonding. Nature 491, 51–55 (2012). 23128225 10.1038/nature11564

[b15] Zhang T. H., Klok J., Tromp R. H., Groenewold J. & Kegel W. K. Non-equilibrium Cluster State in Colloids with Competing Interactions. Soft Matter 8, 667–672 (2012).

[b16] Schade N. B. *et al.* Tetrahedral Colloidal Clusters from Random Parking of Bidisperse Spheres. Phys. Rev. Lett. 110, 148303 (2013). 25167045 10.1103/PhysRevLett.110.148303

[b17] Klix C. L. *et al.* Novel kinetic trapping in charged colloidal clusters due to self-induced surface charge organization. Sci. Rep. 3, 2072 (2013). 23797807 10.1038/srep02072 PMC3691564

[b18] Nakamura Y., Okachi M., Toyotama A., Okuzono O. & Yamanaka J. Controlled Clustering in Binary Charged Colloids by Adsorption of Ionic Surfactants, Langmuir 31, 13303–13311 (2015). 26583431 10.1021/acs.langmuir.5b02778

[b19] Leunissen M. E. *et al.* Ionic colloidal crystals of oppositely charged particles. Nature 437, 235–240 (2005). 16148929 10.1038/nature03946

[b20] Wang J. *et al.* Structural and optical characterization of 3D binary colloidal crystal and inverse opal films prepared by direct co-deposition. J. Mater. Chem. 18, 981–988 (2008).

[b21] Filion L. & Dijkstra M. Prediction of binary hard-sphere crystal structures. Phys. Rev. E 79, 046714-1-9 (2009). 10.1103/PhysRevE.79.046714 19518387

[b22] Dong A., Chen J., Vora P. M., Kikkawa J. M. & Murray C. B. Binary nanocrystal superlattice membranes self-assembled at the liquid–air interface. Nature 466, 474–477 (2010). 20651688 10.1038/nature09188

[b23] Kozina A., Sagawe D., Díaz-Leyva P., Bartsch E. & Palberg T. Polymer-enforced crystallization of a eutectic binary hard sphere mixture. Soft Matter, 8, 627–630 (2012). 10.1039/c7sm90034a 28267177

[b24] Kozina A., Díaz-Leyva P., Palberg T. & Bartsch E. Crystallization kinetics of colloidal binary mixtures with depletion attraction. Soft Matter 10, 9523–9533 (2014). 25354340 10.1039/c4sm02193b

[b25] Joannopoulos J. D., Meade R. D. & Winn J. N. Photonic Crystals. Modeling the flow of light (Princeton University Press, 1995).

[b26] Moon J. H. & Yang S. Chemical Aspects of Three-Dimensional Photonic Crystals. Chem. Rev. 110, 547–574 (2010). 19655793 10.1021/cr900080v

[b27] Asakura S. & Osawa F. On Interaction between Two Bodies Immersed in a Solution of Macromolecules. J. Chem. Phys. 22, 1255–1256 (1954).

[b28] Bartlett P. Fractionated crystallization in a polydisperse mixture of hard spheres. J. Chem. Phys. 109, 10970–10975 (1998).

[b29] Lekkerkerker H. N. & Tuinier R. Colloids and the Depletion Interaction (Springer, Netherland, 2011).

[b30] Leocmach M., Royall C. P. & Tanaka H. Novel zone formation due to interplay between sedimentation and phase ordering. Euro. Phys. Lett. 89, 38006-1-6 (2010).

[b31] Israelachvili. J. Intermolecular and Surface Forces, Chapter 6, Second Edition (Academic Press, London, 1992).

[b32] Vrij A. Polymers at Interfaces and the Interactions in Colloidal Dispersions. Pure Appl. Chem., 48 471–483 (1976).

[b33] Nozawa J. *et al.* Impurity partitioning during colloidal crystallization. J. Phy. Chem. B, 117, 5289–5295 (2013). 10.1021/jp309550y 23544615

[b34] Bibette J. Depletion interactions and fractionated crystallization for polydisperse emulsion purification. J. Colloid Interf. Sci. 147, 474–478 (1991).

[b35] Berthier L. & Witten T. A. Glass transition of dense fluids of hard and compressible spheres. Phys. Rev. E 80, 021502-1-15 (2009). 10.1103/PhysRevE.80.021502 19792128

[b36] Porter D. A., Easterling K. E. & Sherif M. Y. Phase Transitions in Metals and Alloys, 3rd edition, (CRC press, Taylor&Francis, NY, 2009).

[b37] Dutta P. S. In Handbook of Crystal Growth (eds, Dhanaraj G., Byrappa K., Prasad V., Dudley M. ) Ch. 10, 300–302 (Springer, Berlin, 2010).

[b38] Golding R. K., Lewis P. C., Kumacheva E., Allard M. & Sargent E. H. *In situ* study of colloid crystallization in constrained geometry. Langmuir 20, 1414–1419 (2004). 15803727 10.1021/la030145u

[b39] Murai M. *et al.* Unidirectional crystallization of charged colloidal silica due to the diffusion of a base. Langmuir 23, 7510–7517 (2007). 17530873 10.1021/la700754s

[b40] Chonde Y. & Krieger I. M. Emulsion polymerization of styrene with ionic comonomer in the presence of methanol. J. Appl. Polym.Sci. 26, 1819–1827 (1981).

