# Supplementary material for: Spontaneous Formation of Eutectic Crystal Structures in Binary and Ternary Charged Colloids due to Depletion Attraction
Source: Sci Rep. 2016 Mar 17;6:23292. doi: 10.1038/srep23292 (PMC4794737; doi:10.1038/srep23292)
Supplement: Supplementary Information S4 [file srep23292-s4.doc]

**Supplementary Information**

Spontaneous Formation of Eutectic Crystal Structures in Binary and Ternary Charged Colloids due to Depletion Attraction

Akiko TOYOTAMA, Tohru OKUZONO, Junpei YAMANAKA

**Supplementary Information S1**

A movie of the crystal growth process
 (x40 speed, 5 frames per sec. PS600 particles, The particle diamater = 598 nm; Concentration of NaPAA = 0.08 wt%.)

**Supplementary Information S2**

A movie of the crystal growth process
 (5 frames per sec. PS600 particles, The particle diamater = 598 nm; Concentration of NaPAA = 0.08 wt%.)

**Supplementary Information S3**

A movie of the exclusion process
 (15 frames per sec. PS600 + G300 particles, The particle diamaters = 598 nm and 333 nm; Concentration of NaPAA = 0.08 wt%.)

**Supplementary Information S4**

Titration curves of PAA


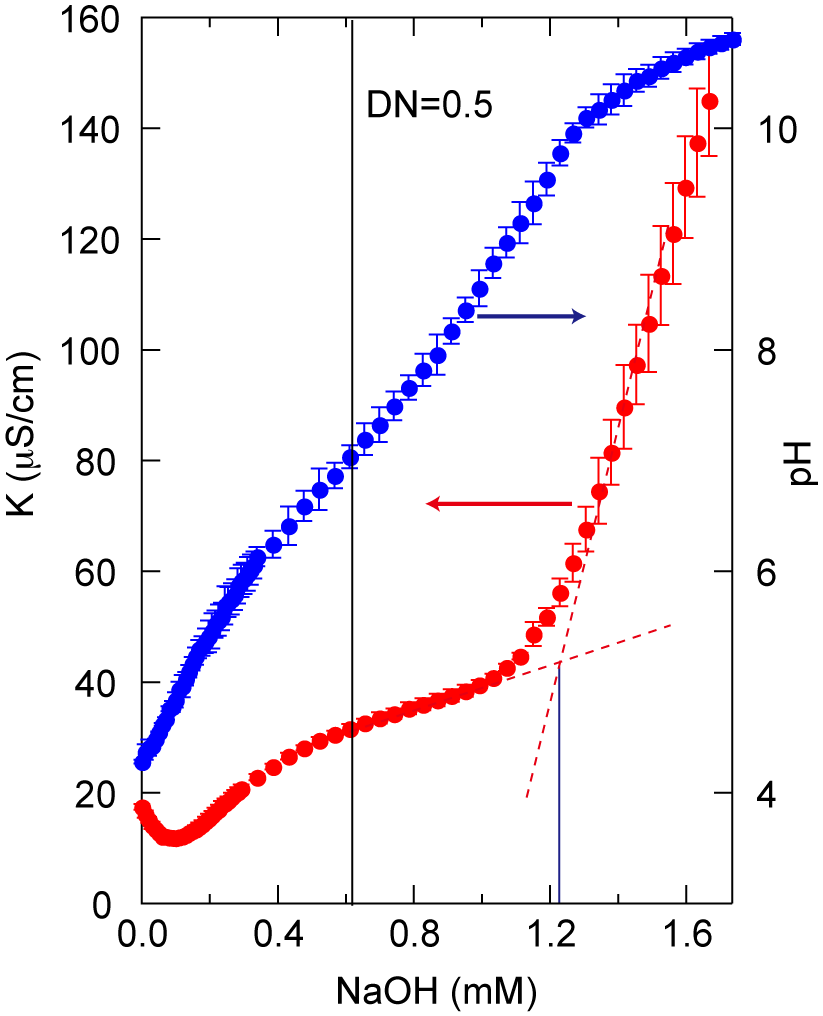


**Figure S1** Conductivity (K, red) and pH (blue symbols) titration curves for aqueous PAA solution by NaOH at 25°C.

Because acrylic acid is a weak acid, PAA is only slightly charged due to partial dissociation of COOH groups (-COOH ↔ -COO^-^ + H^+^) in the absence of alkaline. On addition of alkaline, e.g., NaOH, they are neutralized (-COOH + Na^+^OH^-^  → -COO^-^ + Na^+^ + H_2_O) to generate charges. The degree of dissociation DN is defined as a ratio of molarity of added alkaline to molarity of monomer. At higher DN, the number of charged monomer on a single PAA chain, Z, is larger. Figure S1 shows conductivity and pH titration curves for salt-free aqueous PAA solution at 25°C. Bars are experimental errors in standard deviation of three measurements. The neutralization point determined as an inflection point in the *K* titration curve was 1.23 mM (*C*_p_ = 8.8x10^-3^wt%).

The *K* value of the electrolyte solution is represented by *K* = 10^-3^ Σ(*Λ _i_ C _i_*), where *Λ_i_* and *C _i_* are the molar conductivity and molar concentration of *i*-th ion (H^+^, Na^+^ or PAA^-^ ions). Because *Λ*_H+_* * ( = 350 Scm^2^/mol in water at 25°C) is much larger than *Λ* _PAA-_, we estimated the concentration of free H^+^ ion at DN = 0 by assuming *K* = 10^-3^*Λ*_H+_*C*_H+_. The *C*_H+_ thus estimated was 54 μM, which was very close to *C*_H+_ evaluated from pH (52 μM). The fraction of effective charge, *Z*_eff_/PD, where PD is the polymerization degree (=8.7×10^3^) and *Z*_eff_ the effective charge number on a PAA chain, was 0.04 at DN = 0. At DN = 0.5, *C*_H+_ obtained from pH was negligibly small (0.4μM). By assuming the transport number of Na^+^ in NaPAA solution is 0.5, we estimated *Z*_eff_ = 2200 (*Z*_eff_ /PD = 0.26).
